# Factors Affecting Cognitive Dysfunction Screening for Latinx Adults With Type 2 Diabetes

**DOI:** 10.1093/geroni/igab046.1021

**Published:** 2021-12-17

**Authors:** Heather Cuevas, Julie Zuñiga, Stephanie Morgan

**Affiliations:** 1 The University of Texas at Austin, The University of Texas at Austin/Austin, Texas, United States; 2 The University of Texas at Austin, Austin, Texas, United States

## Abstract

Before development of overt type 2 diabetes (T2DM), changes in brain structure and activation patterns are found in insulin resistance, indicating many with T2DM may already have alterations in cognitive function. How best practices are met for screening for cognitive dysfunction, specifically Latinx adults with T2DM who are at higher risk, remains unclear. The purpose of this study was to examine aspects influencing screening Latinx adults with T2DM for cognitive problems by identifying patient-, clinician- and clinic- level factors. This was a mixed methods study which used semi-structured interviews with Latinx adults with T2DM (n=30; mean age: 68; 57% Mexican American); surveys and interviews with health care providers (n = 15); and inventories of four outpatient clinics to identify factors (e.g. time, clinic policies) influencing screening. Data were analyzed via thematic analysis (interviews) and descriptive statistics (surveys and inventories). For patients, screening was important, but inability to work related to a possible diagnosis of dementia was a concern. Providers and patients agreed other health issues (e.g. hyperglycemia) took precedence to screening. Providers (96.7%) were expected to screen but did not have support/time from clinics and relied on patients for initial prompts. Only one clinic reported staff education on cognitive screening with an emphasis on potential cultural differences in test results and adequate resources related to dementia for Latinx adults. Clinics serving Latinx adults have a responsibility to deliver appropriate care. Leadership should consider innovative practices such creation, with patients, of educational materials for screening -a need highlighted by most participants.

